# Comparison of Bone Bruise Pattern Epidemiology between Anterior Cruciate Ligament Rupture and Patellar Dislocation Patients—Implications of Injury Mechanism

**DOI:** 10.3390/bioengineering10121366

**Published:** 2023-11-28

**Authors:** Ruilan Dai, Yue Wu, Yanfang Jiang, Hongshi Huang, Wenqiang Yan, Huijuan Shi, Qingyang Meng, Shuang Ren, Yingfang Ao

**Affiliations:** 1 Department of Sports Medicine, Peking University Third Hospital, Institute of Sports Medicine of Peking University, Beijing 100080, China; haharuilan@126.com (R.D.); wuyue6063@163.com (Y.W.); anthea_jiang@126.com (Y.J.); huanghs@bjmu.edu.cn (H.H.); 18752009890@163.com (W.Y.); mengqingyang@bjmu.edu.cn (Q.M.); 2 Beijing Key Laboratory of Sports Injuries, Beijing 100080, China; 3 Engineering Research Center of Sports Trauma Treatment Technology and Devices, Ministry of Education, Beijing 100080, China; 4 College of Exercise and Health Sciences, Tianjin University of Sport, Tianjin 300170, China; 5 Biomechanics Laboratory, College of Human Movement Science, Beijing Sport University, Beijing 100080, China; shihuijuan1103@163.com

**Keywords:** anterior cruciate ligament, rupture, patellar dislocation, mechanisms, magnetic resonance imaging, bone bruise pattern

## Abstract

Different bone bruise patterns observed using magnetic resonance imaging (MRI) after non-contact anterior cruciate ligament (ACL) rupture and lateral patellar dislocation may indicate different knee injury mechanisms. In this study, 77 ACL ruptures and 77 patellar dislocations in knee MR images taken from patients with bone bruises at our institution between August 2020 and March 2022 were selected and analyzed. In order to determine typical bone bruising patterns following by ACL rupture and patellar dislocation, sagittal- and transverse-plane images were used to determine bone bruise locations in the directions of medial-lateral and superior-inferior with MR images. The presence, intensity, and location of the bone bruises in specific areas of the femur and tibial after ACL rupture and patellar dislocation were recorded. Relative bone bruise patterns after ACL rupture and patellar dislocation were classified. The results showed that there were four kinds of bone bruise patterns (1-, 2-, 3-, and 4- bone bruises) after ACL rupture. The most common two patterns after ACL rupture were 3- bone bruises (including the lateral femoral condyle and both the lateral-medial tibial plateau, LF + BT; both the lateral-medial femoral condyle and the lateral tibial plateau, BF + LT; and the medial femoral condyle and both the medial and lateral tibial plateau, MF + BT) followed by 4- bone bruises (both the lateral-medial femoral condyle and the tibial plateau, BF + BT), 2- bone bruises (the lateral femoral condyle and tibial plateau, LF + LT; the medial femoral condyle and the lateral tibial plateau, MF + LT; the lateral femoral condyle and the medial tibial plateau, LF + MT; the medial femoral condyle and the tibial plateau, MF + MT; both the lateral-medial tibial plateau, 0 + BT), and 1- bone bruise (only the lateral tibial plateau, 0 + LT). There was only a 1- bone bruise (the latera femoral condyle and medial patella bone bruise) for patellar dislocation, and the most common pattern of patellar dislocation was in the inferior medial patella and the lateral anterior inferior femur. The results suggested that bone bruise patterns after ACL rupture and patellar dislocation are completely different. There were four kinds of bone bruise patterns after non-contact ACL rupture, while there was only one kind of bone bruise pattern after patellar dislocation in patients, which was in the inferior medial patella and lateral anterior inferior femur.

## 1. Introduction

The acute anterior cruciate ligament (ACL) and medial patellofemoral ligament (MPFL) are two important stable structures of the knee. ACL injury results from the knee suddenly changing landing directions, pivoting, or deceleration, while MPFL injury results from not only kinematic forces but also anatomical abnormalities, such as femur or patella morphological abnormality [1,2]. As ACL or MPFL injury will lead to knee pain and functional instability, ACL rupture and patellar dislocation are two of the most considerable knee injury events. So, it is important to explore their injury mechanisms. Previous research has explored the mechanisms of patellar dislocation and ACL rupture, including cadaver studies, numerical simulation studies [3] and video analysis [4,5], which could help to prevent and reduce instances of knee injury, such as ACL rupture and patellar dislocation [6,7,8]. Though these methods provide valuable information for an understanding of the motions leading to ACL rupture and patellar dislocation, the knee joint moves in a three-dimensional space of six degrees of freedom; thus, video analysis and simple computer models may not accurately simulate the real motion trajectory of a knee joint injury. Therefore, it is necessary to find a performance that can directly reflect the injury mechanism of ACL rupture and patellar dislocation.

Traumatically related, irregular areas of reduced signal intensity with T1-weighted images and increased intensity on T2-weighted images after a force on the bone or a bone-to-bone impact are defined as bone bruises [9]. Bone bruises are commonly observed with magnetic resonance imaging (MRI) following ACL rupture or patellar dislocation. As the distribution of the bone bruise is like a footprint left behind by the injury, it can provide an insight into the mechanisms of knee injuries, and the location patterns and severity of bone bruises in patients may indicate the primary mechanisms of non-contact ACL rupture and patellar dislocation [1,10].

In as early as the 1990s, sensitivity intraosseous signal abnormality was found in femoral and tibial bones after ACL rupture by JH Mink, and the medial patellar and lateral femoral after patellar dislocation by E lance [11,12], suggesting that axial MRI can be a valuable adjunct to the evaluation of an acutely injured knee. Previous studies have also shown that observing bone bruises of the knee with MRI could have implications for developing an understanding of the mechanisms of ACL and patellar dislocation injuries [10,11,13]. In addition, the femoral and tibial bone bruise patterns after ACL injury differed to the femur and patella bone bruises in patellar dislocation [14,15], Simple ACL rupture bone bruise exists in the femur and (or) tibia, while simple patella dislocation bone bruise exists in the femur and (or) patella because of their different injury mechanisms. Moreover, the distribution of bone bruise caused by ACL rupture is different. Bone bruises occur in both the medial and lateral of the femur and tibia after ACL rupture, suggesting that the knee flexion, and the tibia rotation angle may be different in different people, as the femur bone bruise patterns of patella dislocation patients suggests, and there are actually limited reports that have compared the bone bruise patterns between different knee injuries.

Therefore, the purpose of this retrospective study was to explore the different bone bruise patterns by analyzing the MRI of ACL ruptures and patellar dislocations, which can assist us to better study not only the performance patterns of bone bruises but also the non-contact sports injury mechanisms of ACL ruptures and patellar dislocations. We hypothesized that the bone bruise patterns of ACL rupture and patellar dislocation were completely different, and that there were only lateral femur and medial patellar bone bruises in patellar dislocation while there were both medial and lateral bone bruises of the femur and tibia in ACL rupture.

## 2. Materials and Methods

### 2.1. Selection Criteria and MR Imaging

Institutional ethics committee approval was acceded for the retrospective study (IRB00006761-M2023142). MR images of 77 ACL rupture and 77 patellar dislocation patients were captured at our institution between August 2020 and March 2022, and those that met the inclusion and exclusion criteria were included in the study. The inclusion criteria were (1) ACL rupture or patellar dislocation was confirmed by an experienced radiologist and a sport medicine doctor with the help of MRI; (2) MRI acquired no more than 1 month after ACL rupture and patella dislocation; (3) trauma related to non-contact activity; and (4) patients aged from 18 to 40 years. The 1-month threshold was implemented as far as possible to reduce timing errors, and make sure the signal characteristics of bone marrow edema were consistent [16]. Only patients aged from 18 to 40 years were selected because bone mineral density gradually reduces in injuries of patients over 40/50 years old, whose signal characteristics of bone contusion could be affected [17,18]. The exclusion criteria were (1) contact injury; (2) other injuries in addition to patella dislocation or ACL rupture; (3) medial or vertical patellar dislocation; and (4) unavailable or unclear MRI.

### 2.2. Determination of Bone Bruise Location

Standard scans were acquired using a 1.5-T scanner (SIGNA (Innsbruck, Austria); GE Healthcare (Chicago, IL, USA)) with a 3.5 mm slice space and 512 × 512 matrix in the sagittal and transverse planes of the injured knee of ACL rupture or patellar dislocation patients. Image slices of the sagittal and transverse sections were used to ensure the bone bruise areas in the medial-lateral and superior-inferior directions, respectively. First, we classified the participants according to whether they were with or without bone bruise; then, the hand-feel region of interest drawing function by ImageJ software (National Institutes of Health, Bethesda, MD, USA, 1.52i) was used to trace the bone bruise borders, and the bone bruise gray values were recorded using imaging pixels. The signal intensity could reflect the impact of bone bruise because the gray value of pixels was obtained according to the MRI signal intensity of the voxel represented in MR images. The maximum gray value of the pixels and the boundary area were measured using ImageJ. The gray value was used to determine how serious of a bone bruise was within an imaging slice in this study. The larger the gray value, the greater the intensity of signal, and the more serious the bone bruise, considering maximum gray value as the site of bone impact. When the same maximum gray value showed on different slices, the level of the bone bruise was further determined according to the area.

**Figure 1:**
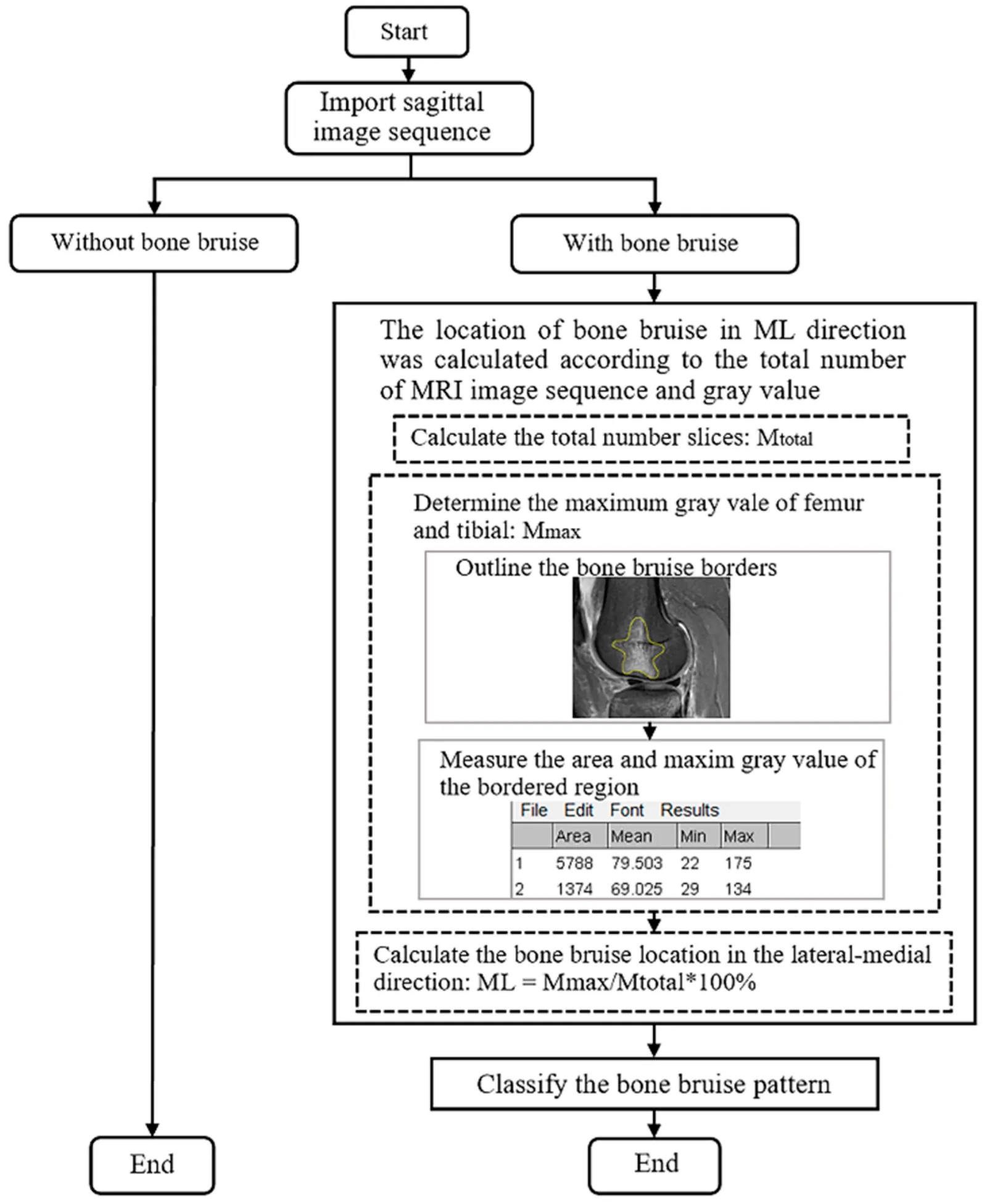
Method of calculating the location of a bone bruise to classify the bone bruise pattern of ACL rupture in sagittal plane. *, represents multiplication.

**Figure 2:**
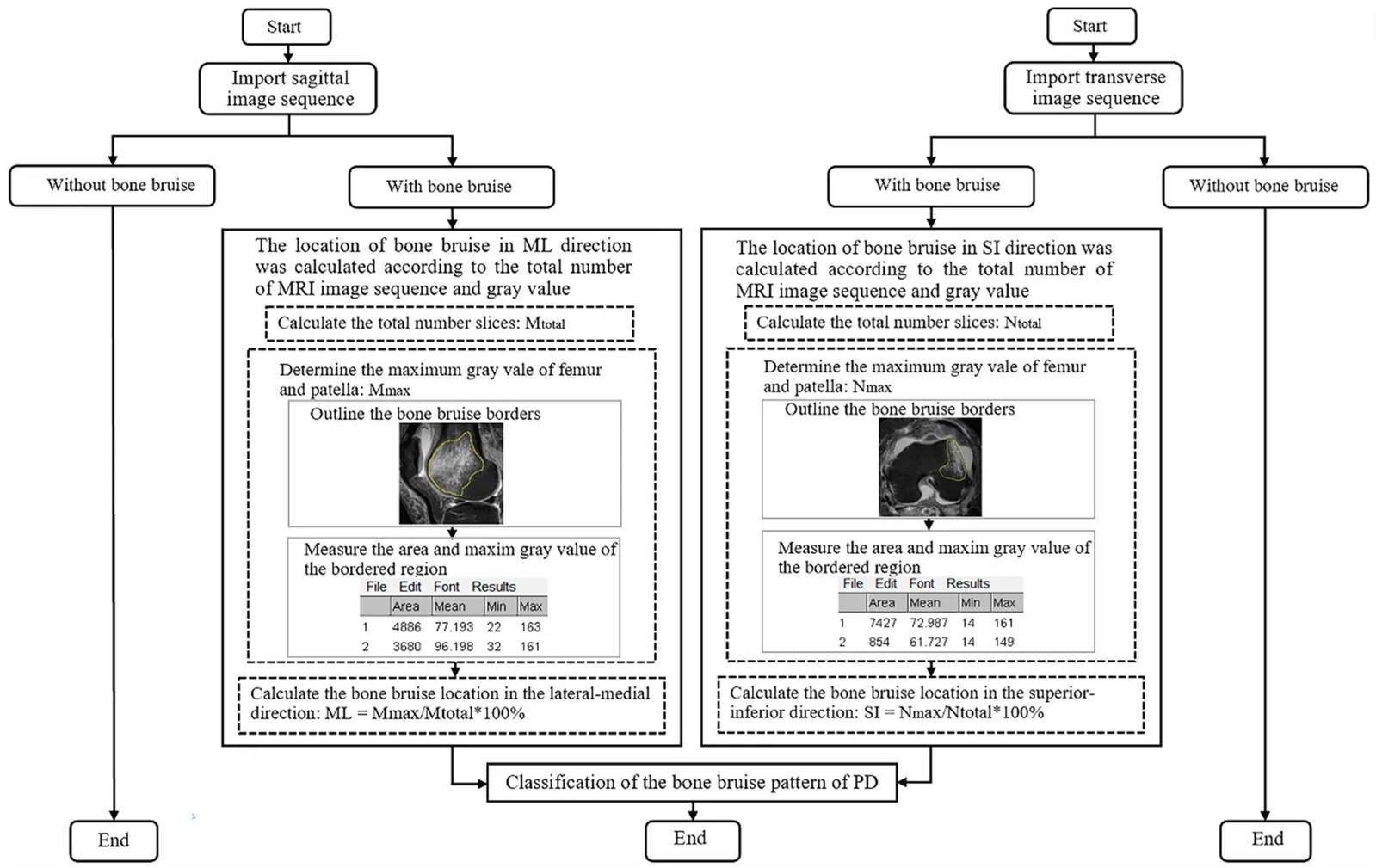
Method of calculating bone bruise location to classify the bone bruise pattern of patellar dislocation in sagittal and transverse planes. *, represents multiplication.

Sagittal- and transverse- plane imaging slices were used to determine the location of bone bruises in the lateral-medial and superior-inferior directions (Figure 1 and Figure 2). The precise locations in the lateral-medial direction were confirmed using the following formula [19]:

(1) $$\mathrm{ML}=M_{max}/M_{total}\times 100\%$$

*M_max_* is defined as the number of slices at the maximum gray value of all the slices in the order of lateral-medial direction in the sagittal plane while *M_total_* is the total number of slices in the sagittal plane based on the slice of bone segments. ML is the measurement of the position in the medial-lateral direction which ranges from 0% (closer to the lateral condyle of the femur or patellar) to 100% (closer to the medial condyle of the femur or patellar).

The method of determining the bone bruise location in the superior-inferior direction was similar to that used for determining those on lateral-medial sides of the bone, which uses the following formula:

(2) $$\mathrm{SI}=N_{max}/N_{total}\times 100\%$$

*N_max_* is defined as the number of slices at the maximum gray value among all the slices on the superior-inferior sides in the transverse plane, whereas *N_total_* is the total number of slices in the transverse plane based on the bone segments. SI is used to measure the location in the superior-inferior direction, and it ranges from 0% (closer to the superior side of the femoral trochlea or patellar) to 100% (closer to the inferior side of the femoral trochlea or patellar). Selecting the image of the largest anterior and posterior diameter of the femoral condyle as the reference, a bone bruise in the femur’s most superior end was defined as the location where three slices (10.5 mm) are from the horizontal tangent of the femoral posterior condyle cartilage margin, and bone bruise in the femur’s most inferior end was defined as where the horizontal tangent is parallel to the bottom of the femoral condyle in the sagittal plane.

### 2.3. Statistical Analysis

The Kolmogorov–Smirnov test was used to evaluate whether the data conformed to a normal distribution. Continuous data were described as the mean ± standard deviation (SD), whereas non-normal distribution data were expressed as the mean with interquartile range, M (Q1, Q3). Categorical data were described as a number. Unpaired Student’s *t* tests were used to detect differences between the ACL rupture and dislocation groups in the continuous variables. Mann–Whitney tests were used to detect differences between the non-normal distribution data for the continuous and categorical variables (age, time from injury, sex, and injury causes) of the ACL rupture and dislocation groups. A *p*-value of <0.05 was considered statistically significant.

## 3. Results

### 3.1. Descriptive Statistics

MR images of 77 ACL ruptures (63 men [81.8%], 14 women [18.2%], aged 29.06 ± 6.09 years) and 77 patellar dislocations (32 men [41.6%], 45 women [58.4%], aged 24.83 ± 6.02 years) in patients at our center who met the inclusion criteria were recorded in this study. The causes of ACL rupture were all sports related, while nearly half of the patellar dislocations were caused by daily activities. The epidemiology of the patients, namely an analysis of their age, BMI, sex, and injury-causing events, was significantly different between the ACL rupture and patellar dislocation groups; ACL rupture patients were significantly older than patellar dislocation patients, (Table 1). Bone bruises caused by ACL rupture were all in the femur and (or) tibia, while those caused by patellar dislocation were all in femur and patella (Table 1 and Table 2).

### 3.2. Bone Bruises Patterns of ACL Rupture and Patellar Dislocation

**Figure 3:**
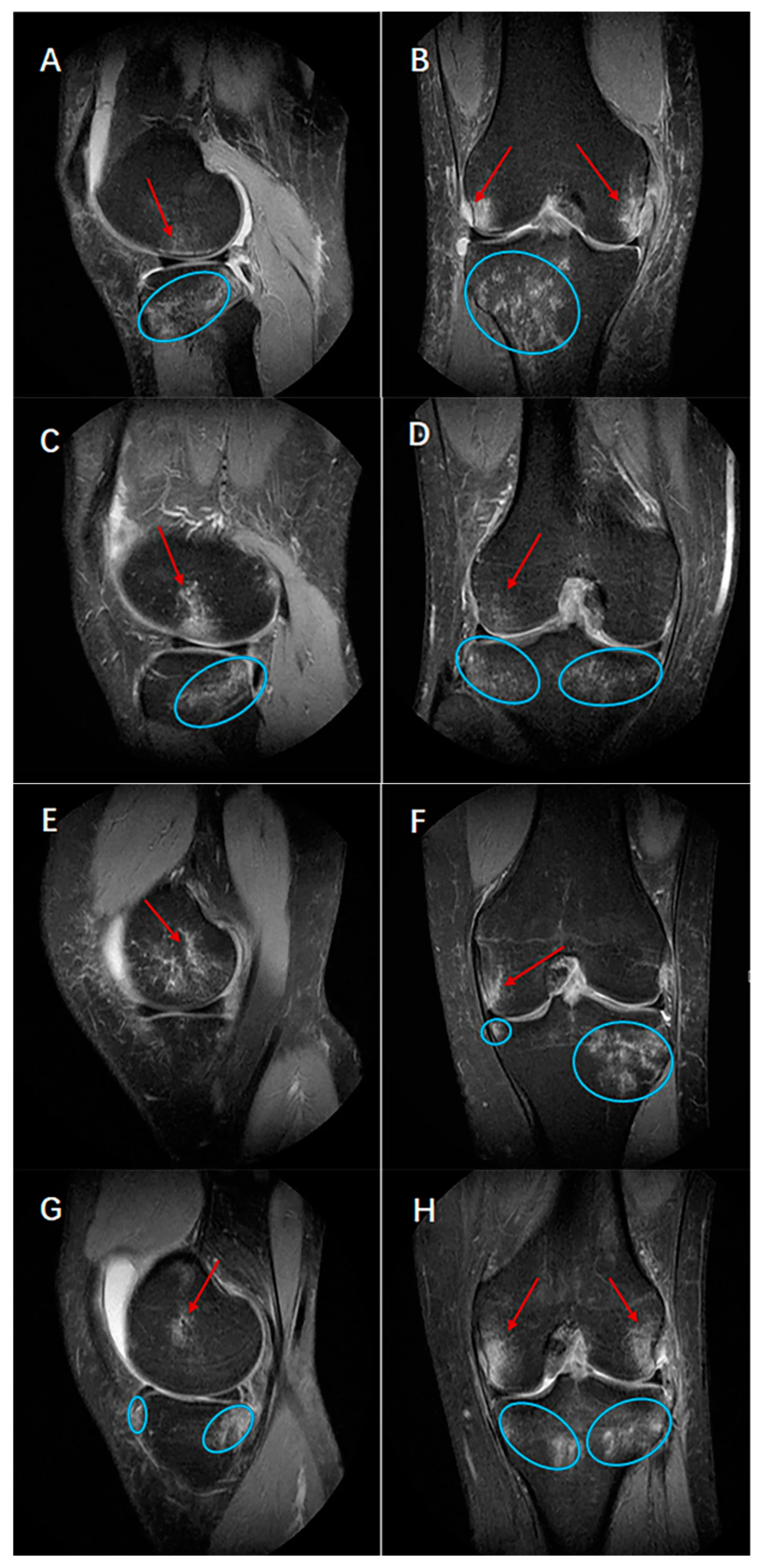
The most common 3- (**A**–**F**) and 4- (**G**,**H**) bone bruise patterns for ACL rupture. (**A**,**B**), the lateral femoral condyle and both the medial and lateral tibial plateau (LF + BT). (**C**,**D**), both the lateral and medial femoral condyle and lateral tibial plateau (BF + LT). (**E**,**F**), the medial femoral condyle and both the lateral and medial tibial plateau (MF + BT). (**G**,**H**) both the lateral and medial femoral condyle and tibial plateau (BF + BT). (**A**,**C**,**E**,**G**), sagittal plane. (**B**,**D**,**F**,**H**), coronal plane. Red arrow, femoral bone bruise. Blue ellipse, tibia bone bruise.

By calculating the position of bone bruises in the sagittal and coronal planes, we determined there were four kinds of bone bruise (1-, 2-, 3-, 4- bone bruises) patterns among the ACL rupture patients (Table 3). The most common two patterns of ACL rupture were three (40%) bone bruises (the lateral femoral condyle and both the lateral and the medial tibial plateau, LF + BT (23%); both the lateral and medial femoral condyle and the lateral tibial plateau, BF + LT (16%); the medial femoral condyle and both the lateral and medial tibial plateau, MF + BT (1%)), followed by four (29%) (both the lateral and medial femoral condyle and tibial plateau, BF + BT) (Figure 3A–H) and two (30%) bone bruises (the lateral femoral condyle and tibial plateau, LF + LT (19%); the medial femoral condyle and the lateral tibial plateau, MF + LT (5%); the lateral femoral condyle and the medial tibial plateau, LF + MT (4%); the medial femoral condyle and tibial plateau, MF + MT (1%); the lateral and medial tibial plateau, 0 + BT (1%)). Just one bone bruise included the lateral tibial plateau (0 + LT, 1%).

**Figure 4:**
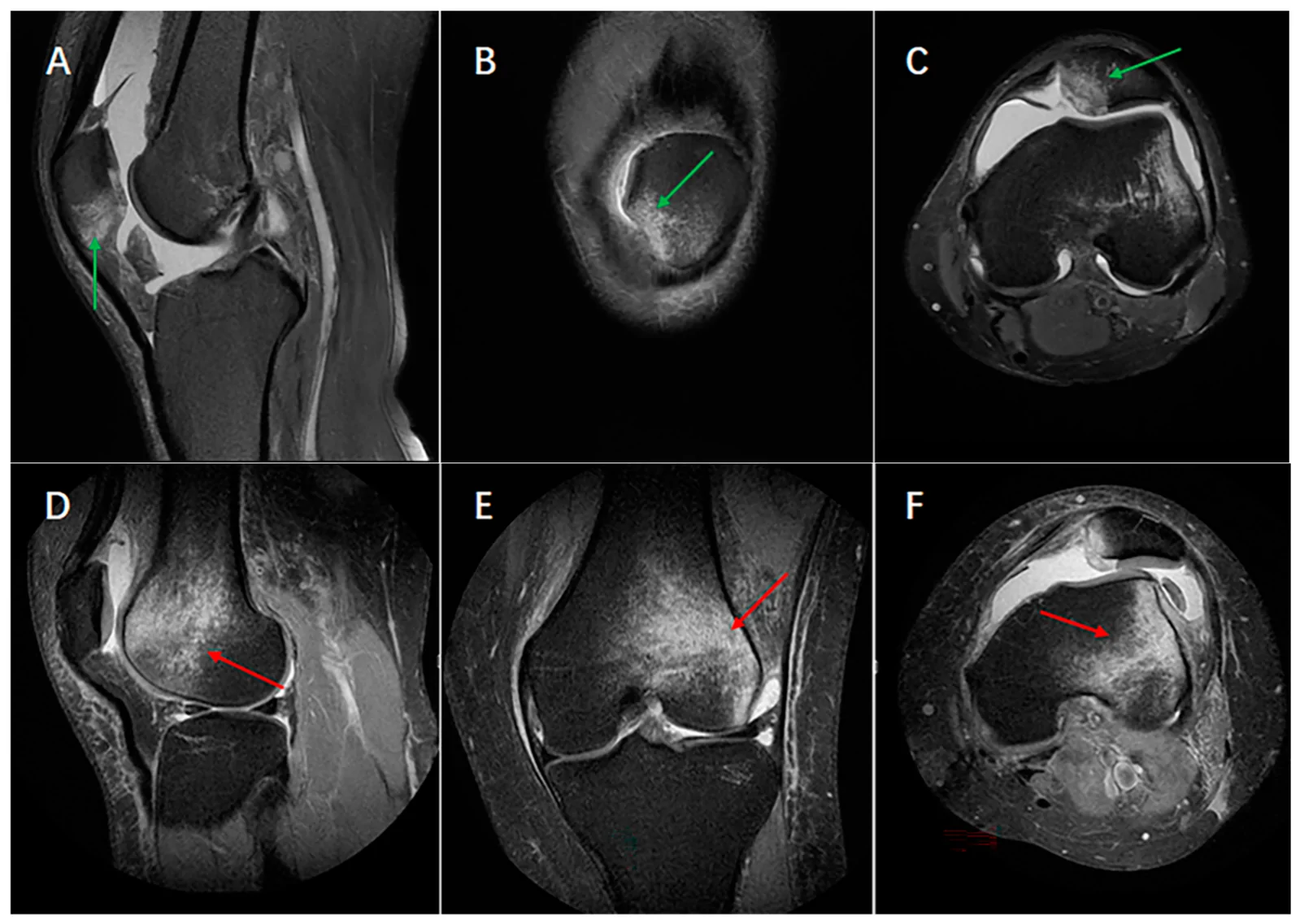
Distribution of patellar bone bruises in three planes of patellar dislocation patients. (**A**,**D**), sagittal plane; (**B**,**E**), coronal plane; (**C**,**F**), transverse plane. Red arrow, femoral bone bruise. Green arrow, patella bone bruise.

By calculating the position of bone bruises in the sagittal and transverse planes, we determined there was only one bone bruise pattern (the lateral femur and medial patella bone bruise) for patellar dislocation (Figure 4). The bone bruise was in the inferior medial patella and lateral anterior inferior femur (Table 3), and seven categories of bone bruise characteristics were obtained as follows: MIP + LAIF (medial inferior side of the patellar and lateral anterior inferior of the femoral condyle, 57%); MIP + LPIF (medial inferior side of the patellar and the lateral posterior inferior of the femoral condyle, 22%); MIP + LASF (medial inferior side of the patellar and lateral anterior superior of the femoral condyle, 10%); MSP + LAIF (medial superior side of the patellar and the lateral anterior inferior of femoral condyle, 6%); MIP + LPSF (medial inferior side of the patellar and lateral posterior superior of the femoral condyle, 1%); MSP + LASF (medial superior side of the patellar and lateral anterior superior of the femoral condyle, 1%); and MSP + LPIF (medial superior side of the patellar and the lateral posterior inferior of femoral condyle, 1%).

## 4. Discussion

Our purpose was to summarize, and compare the different bone bruise patterns observed between ACL rupture and patellar dislocation injuries in this study. Previous studies already reported that there were femur and tibial bone bruises for ACL rupture injuries and patellar and femur bone bruises for patellar dislocation injuries [7,9,10,20,21]. However, the bone bruise patterns were not systematically counted, and there was no comparison between ACL rupture and patellar dislocation injuries. In total, four types of bone bruise patterns after ACL rupture and one type after patellar dislocation were counted and classified in our study, which can help us to understand their different epidemiology characteristics and injury mechanisms (Table 2 and Table 3).

Methods were used to explore the mechanisms of knee joint injuries. Relatively consistent bone bruise patterns were revealed with MRI and video analysis in professional football players with acute ACL knee injuries [22]. A bone bruise is a phenomenon caused by bone–bone impact or by the bone suffering a heavy on-sided strain, observed in MR images as decreased and increased signal intensities in T1- and T2-weighted scans, respectively [9]. Studies have reported femur and patellar bone bruise locations of patellar dislocation patients, as well as femur and tibia bone bruises of ACL injury patients [7,23,24,25]; however, the location and frequency of those in the coronal section were reported separately and did not combine the sagittal and cross-sectional locations in patients. Furthermore, the femur and patella as well as tibial bones are all three-dimensional structures [3], and the locations of bone bruises in their various sections provide valuable clues as to the associated injuries, which would help in understanding the mechanisms of ACL rupture and patellar dislocation [13,26]. Therefore, we hypothesized that the bruise patterns of ACL rupture and patellar dislocation injuries were completely different, and also that there are only lateral femur and medial patella bone bruises in patellar dislocation, while there are both medial and lateral bone bruises of the femoral condyle and tibial plateau in ACL rupture.

To test our hypotheses, we reviewed the MR images of ACL rupture and patellar dislocation patients with bone bruises. Imaging slices of the sagittal and transverse sections were used to quantitatively calculate the relative lateral-medial and superior-inferior direct positions of bone bruises in the distal femur, proximal tibia, and patella. As shown in our study, there were four (1-, 2-, 3-, 4-) kinds of bone bruise patterns for ACL rupture, and the most commonly seen bone bruise patterns were 3- (LF + BT, BF + LT, MF + BT), followed by 4- (BF + BT) and 2- bone bruises (LF + LT, MF + LT, LF + MT, MF + MT, 0 + BT; Table 2) in the femur and tibia, while there was only one bone bruise pattern for patellar dislocation, which was in the lateral femur and medial patella (Figure 1 and Figure 2, Table 3). ACL rupture and patellar dislocation injuries had completely different bone bruise patterns, which may represent the different injury mechanisms of the knee. The ACL rupture patients displayed 3- or 4- bone bruises with either 1 or 2 femoral, bone bruises and 2 tibial bone bruises, suggesting that the knee joint was unstable when the ACL was injured, not only in the anterior, which translated the tibia to the femur, but also through obvious shaking in the medial-lateral direction at the time of the trauma to the knee. Similarly, the differences in the superior-inferior direct positions of femoral and patellar bone bruises in patellar dislocation also indicated that different bone bruise patterns reflect different knee flexion angles at the time of injury (Figure 4).

**Figure 5:**
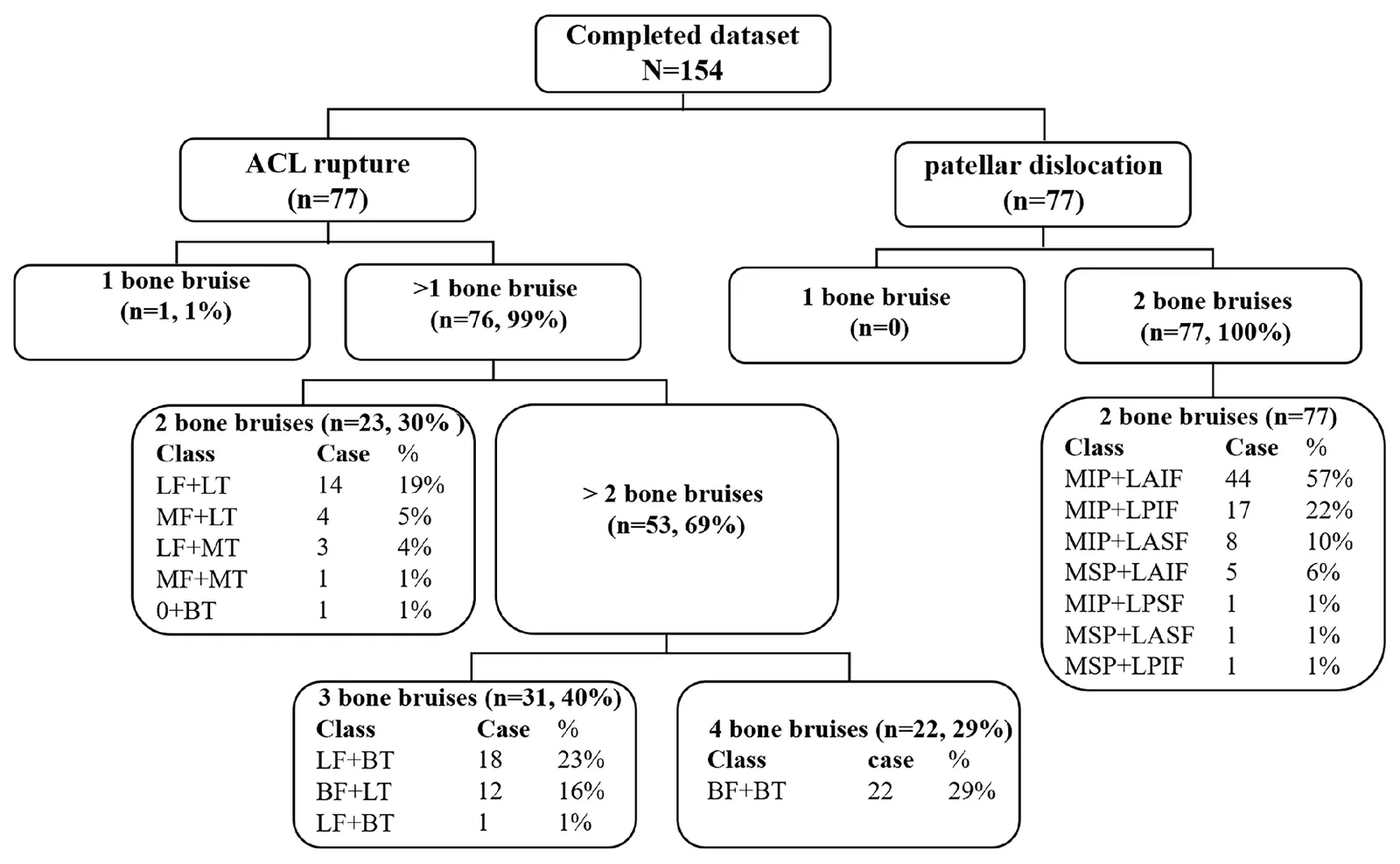
Classification tree of bruise patterns of ACL rupture and patellar dislocation. LF + BT, BF + LT, MF + BT, BF + BT, LF + LT, MF + LT, LF + MT, MF + MT, 0 + BT, 0 + LT, MIP + LAIF, MIP + LPIF, MIP + LASF, MSP + LAIF, MIP + LPSF, MSP + LASF, MSP + LPIF, and the annotation seen above.

Despite many studies on the location of bone bruises, they have only pointed out the characteristics of the bone bruises, and there was no description of the specific location, nor has there been a systematic report on the pattern classification of bone bruises after ACL rupture and patellar dislocation [10,23,25]. Our study not only recorded the bone bruises but also calculated the specific locations of bone bruises after ACL rupture and patellar dislocation (Figure 5). For non-contact-sport knee injuries, a bone bruise is not only an intensity signal but also a footprint of the damage process, which could provide information about the injury mechanism or its product, because different mechanisms of injury have different bone bruise patterns [14]. Numerical optimization was used by Kim Sophia Y et al. to maximize the overlap of the bone bruises on the femur and tibia and to predict the position of injury for the most common 3- and 4- bone bruises for ACL rupture, establishing an infrastructure to create three-dimensional surface models. They found that landing on an extended knee results in a high risk of ACL injury [7,20], while others found that the knee valgus is a critical component of the injury mechanism [5,27,28]. However, the initial injury mechanism of 3- and 4- bone bruises after ACL rupture was different. Our study found that the bone bruise patterns of ACL rupture and patellar dislocation injuries were completely different. This is related to their different anatomical structures because ACL rupture is caused by an instantaneous impact of the femur and tibial bones, while patellar dislocation is caused by injury of the medial patellofemoral ligament, partly due to the abnormal anatomy of the patella and femur [29,30].

Although the bone bruise patterns were completely different, they represent their own sports injury mechanisms. It was found that the video analysis was consistent with the pattern of bone bruising, which showed that it was feasible and scientific to analyze the injury mechanism according to the bone bruise, and it was also mentioned that although they constitute the same kind of injury, different bone bruise patterns may represent different sports injury mechanism patterns [22,31,32]. A more scientific and targeted model of combined specific bone bruise manifestation needs to be established to explore the mechanisms of non-contact sports injuries [33].

In addition, the characteristics of the patient cohort, including age, sex, and injury-causing events, were significantly different between the ACL rupture and patellar dislocation groups (Table 1). The age of the patellar dislocation patients was significantly younger than that of ACL rupture patients, and there were more men (81.8%) in the ACL rupture group, while there were more women (58.4%) in the patellar dislocation group. Although the rate of ACL rupture was three times higher in men [34], the results of this study take into consideration that this was due to the higher total number of men participating in high-level sports, producing an increased number of people encountering tibial forward movement as well as landing torsion, which are the risk factors for ACL injury [35]. In our study, the causes of ACL rupture were all sports (100%), while sporting causes of patellar dislocation only accounted for 54.5%, and the remaining 45.5% resulted from daily activities. As for patellar dislocation patients, almost all of them had anatomical abnormalities [36,37], which is more common among women, especially in children, resulting in the patella slipping to the outside of the knee joint more easily, not only in sports activities, but also during everyday-life knee flexion and torsion motion [38,39,40].

**Figure 6:**
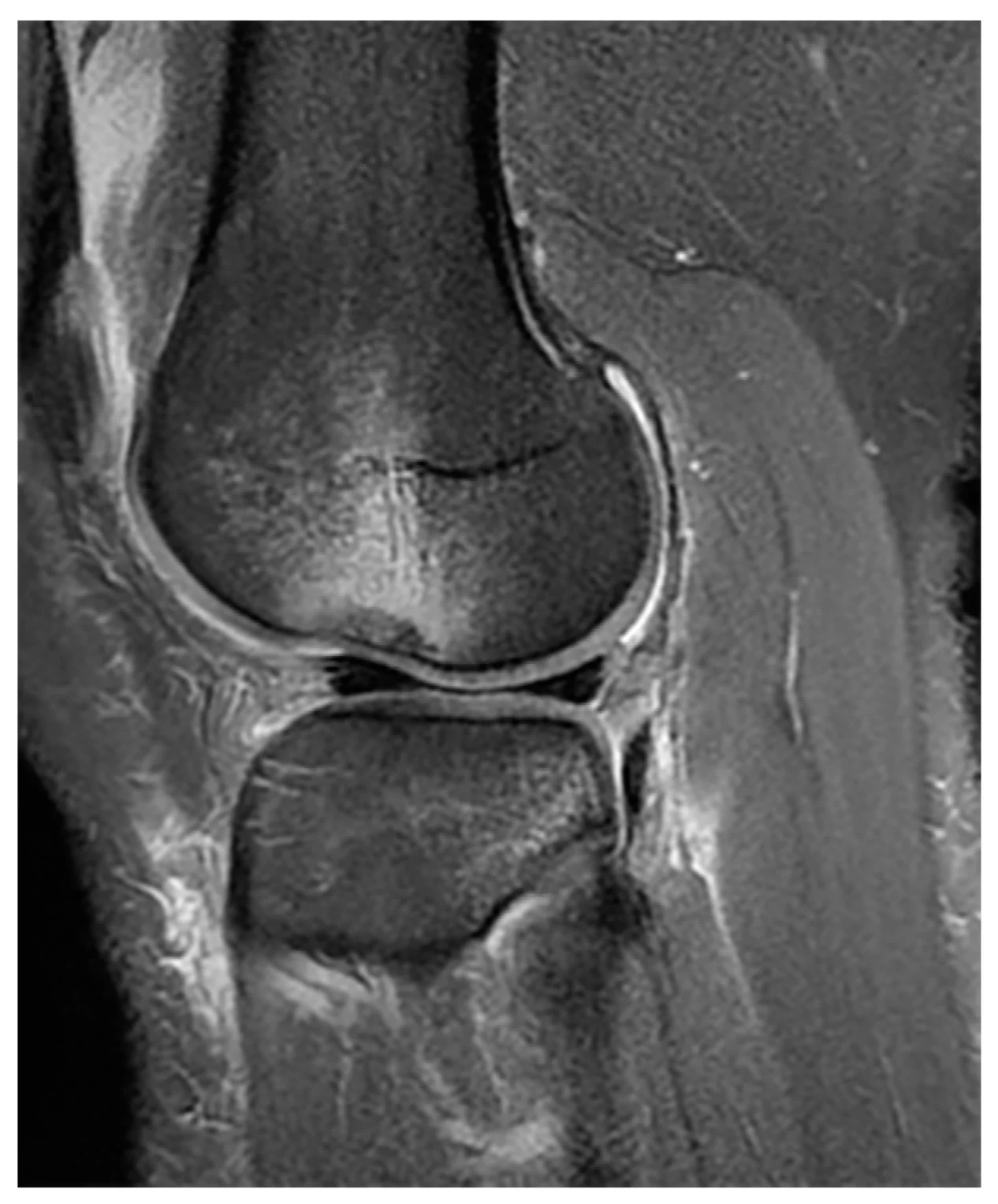
Lateral femoral notch sign on sagittal T2-weighted magnetic resonance imaging in an anterior cruciate ligament injury.

In addition to the above characteristics, we also found an interesting phenomenon. In the ACL injury patients, the sagittal MR images accounting for 90% of the images (Figure 6) in our study had an obvious lateral femoral notch (LFN), which didn’t exist in patellar dislocation patients. A LFN is a radiological feature observed in the MRI in the sagittal plane of the lateral femoral condyle. The LFN depth may lead to a decrease in the contact area between the distal femur and the proximal tibia, and may result in altered knee kinematics and instability. However, how the LFN affects the knee kinematics actually was still unclear. Ajay et al. indicated that LFN depth was not associated with increased rotatory instability [41], while Gian et al. hold that LFNs are correlated with increased rotatory laxity after ACL injury [42]. But they all agreed with the idea that an LFN is a concomitant of posterior root tears of the lateral meniscus [43]. The relationship between LFNs and the femur and tibia, especially when valgus stresses are applied, along with external tibial rotation should be further explored in future research. For patellar dislocation patients, the location of bone bruise was on the lateral condyle of the superior or inferior femur, which was also very interesting. Since the accepted risk knee angle of patellar dislocation was flexion of 20–30 degrees, the inferior femur bone bruise suggests that the knee joint experienced an enlarged knee flexion angle when patellar dislocation occurred, which should be also further explored in future research.

### Limitation

There are several limitations of our study. First, the sample size was too small to reflect the difference between ACL rupture and patellar dislocation with patient information and bone bruise patterns. Second, we did not compare the anatomical indexes between the ACL rupture and patellar dislocation patients.

## 5. Conclusions

The patterns of bone bruising after ACL rupture and patellar dislocation were completely different. There were four kinds (1-, 2-, 3-, 4- bone bruises) of bone bruises in the femur and tibial bones of ACL patients, while there was only one kind of bone bruise pattern for patellar dislocation in the inferior medial patella and lateral anterior inferior femur. The epidemiology of ACL rupture and patellar dislocation was also quite different, and ACL rupture patients were significantly older than patellar dislocation patients. There were more women than men in the patellar dislocation group, and the opposite was true for ACL rupture patients.

## Figures and Tables

**Table 1:** Patient information.

	ACL Rupture	Patellar Dislocation	*p* Value
Age	29.06 ± 6.09	24.83 ± 6.02	<0.001
BMI ^a^	23.98 ± 3.29	23.50 ± 3.67	0.404
Time from injury (d)	19.22 ± 6.40	14.45 ± 8.66	<0.001
Sex, man/woman, n	63/14	32/45	0.001
Injury side, left/right, n	43/34	49/28	0.326
Causing sports/daily activities, n	77/0	42/35	<0.001

^a^ BMI, Body Mass Index.

**Table 2:** Bone bruise patterns of ACL rupture ^a^.

	Bone Bruise Pattern	N (%)
3- bone bruises		
	LF + BT	18 (23%)
	BF + LT	12 (16%)
	MF + BT	1 (1%)
4- bone bruises		
	BF + BT	22 (29%)
2- bone bruises		
	LF + LT	14 (19%)
	MF + LT	4 (5%)
	LF + MT	3 (4%)
	MF + MT	1 (1%)
	0 + BT	1 (1%)
1- bone bruise		
	0 + LT	1 (1%)
Total		77 (100%)

^a^ There were 4 kinds (10 categories) of bone bruise patterns for ACL rupture in femur and tibial bones, as follows: LF + BT, the lateral femoral condyle and both the lateral and medial tibial plateau; BF + LT, both the lateral and medial femoral condyle and lateral tibial plateau; MF + BT, the medial femoral condyle and both the lateral and medial tibial plateau; BF + BT, both the lateral and medial femoral condyle and tibial plateau; LF + LT, the lateral femoral condyle and tibial plateau; MF + LT, the medial femoral condyle and the lateral tibial plateau; LF + MT, the lateral femoral condyle and the medial tibial plateau; MF + MT, the medial femoral condyle and tibial plateau; 0 + BT, only both the lateral and medial tibial plateau; and 0 + LT, only the lateral tibial plateau.

**Table 3:** Bone bruise patterns of patellar dislocation ^b^.

Bone Bruise Pattern	N (%)
MIP + LAIF	44 (57%)
MIP + LPIF	17 (22%)
MIP + LASF	8 (10%)
MSP + LAIF	5 (6%)
MIP + LPSF	1 (1%)
MSP + LASF	1 (1%)
MSP + LPIF	1 (1%)
Total	77 (100%)

^b^ There was only 1 kind (7 categories) of bone bruise pattern for patellar dislocation, which only occurred in the lateral femur and medial patella, as follows: MIP + LAIF, medial inferior side of the patellar and lateral anterior inferior of the femoral condyle; MIP + LPIF, medial inferior side of patellar and lateral posterior inferior of the femoral condyle; MIP + LASF, medial inferior side of the patellar and lateral anterior superior of the femoral condyle; MSP + LAIF, medial superior side of the patellar and lateral anterior inferior of the femoral condyle; MIP + LPSF, medial inferior side of the patellar and lateral posterior superior of the femoral condyle; MSP + LASF, medial superior side of the patellar and lateral anterior superior of the femoral condyle; and MSP + LPIF, medial superior side of the patellar and lateral posterior inferior of the femoral condyle.

## Data Availability

The data of this study were included in the article.

## Abbreviations

MRI	Magnetic resonance imaging
ACL	Anterior cruciate ligament
BMI	Body mass index
SD	Mean ± standard deviation
LF + BT	The lateral femoral condyle and both medial and lateral tibial plateau
BF + LT	Both the lateral-medial femoral condyle and lateral tibial plateau
MF + BT	The medial femoral condyle and both lateral and medial tibial plateau
BF + BT	Both the lateral-medial femoral condyle and tibial plateau
LF + LT	The lateral femoral condyle and tibial plateau
MF + LT	The medial femoral condyle and lateral tibial plateau
LF + MT	The lateral femoral condyle and medial tibial plateau
MF + MT	The medial femoral condyle and tibial plateau
0 + BT	Only both the medial and lateral tibial plateau
0 + LT	Only the Lateral tibial plateau
MIP + LAIF	Medial inferior side of the patellar and the lateral anterior inferior femoral condyle
MIP + LPIF	Medial inferior side of the patellar and the lateral posterior inferior femoral condyle
MIP + LASF	Medial inferior side of the patellar and the lateral anterior superior femoral condyle
MSP + LAIF	Medial superior side of the patellar and the lateral anterior inferior femoral condyle
MIP + LPSF	Medial inferior side of the patellar and the lateral posterior superior femoral condyle
MSP + LASF	Medial superior side of the patellar and the lateral anterior superior femoral condyle
MSP + LPIF	Medial superior side of the patellar and the lateral posterior inferior femoral condyle

## References

[1] Qiu L., Sheng B., Li J., Xiao Z., Yuan M., Yang H., Lv F., Lv F. (2021). Mechanisms of non-contact anterior cruciate ligament injury as determined by bone contusion location and severity. Quant. Imaging Med. Surg..

[2] Kim H.K., Parikh S. (2022). Patellofemoral Instability in Children: Imaging Findings and Therapeutic Approaches. Korean J. Radiol..

[3] Spang R.C., Jahandar A., Meyers K.N., Nguyen J.T., Maher S.A., Strickland S.M. (2021). Dysplastic Patellofemoral Joints Lead to a Shift in Contact Forces: A 3D-Printed Cadaveric Model. Am. J. Sports Med..

[4] Koga H., Nakamae A., Shima Y., Bahr R., Krosshaug T. (2018). Hip and Ankle Kinematics in Noncontact Anterior Cruciate Ligament Injury Situations: Video Analysis Using Model-Based Image Matching. Am. J. Sports Med..

[5] Koga H., Nakamae A., Shima Y., Iwasa J., Myklebust G., Engebretsen L., Bahr R., Krosshaug T. (2010). Mechanisms for noncontact anterior cruciate ligament injuries: Knee joint kinematics in 10 injury situations from female team handball and basketball. Am. J. Sports Med..

[6] Farahmand F., Tahmasbi M.N., Amis A. (2004). The contribution of the medial retinaculum and quadriceps muscles to patellar lateral stability—An in-vitro study. Knee.

[7] Kim S.Y., Spritzer C.E., Utturkar G.M., Toth A.P., Garrett W.E., DeFrate L.E. (2015). Knee Kinematics During Noncontact Anterior Cruciate Ligament Injury as Determined From Bone Bruise Location. Am. J. Sports Med..

[8] Marchetti D.C., Moatshe G., Phelps B.M., Dahl K.D., Ferrari M.B., Chahla J., Turnbull T.L., LaPrade R.F. (2017). The Proximal Tibiofibular Joint: A Biomechanical Analysis of the Anterior and Posterior Ligamentous Complexes. Am. J. Sports Med..

[9] Kaplan P.A., Gehl R.H., Dussault R.G., Anderson M.W., Diduch D.R. (1999). Bone contusions of the posterior lip of the medial tibial plateau (contrecoup injury) and associated internal derangements of the knee at MR imaging. Radiology.

[10] Sanders T.G., Medynski M.A., Feller J.F., Lawhorn K.W. (2000). Bone contusion patterns of the knee at MR imaging: Footprint of the mechanism of injury. RadioGraphics.

[11] Lance E., Deutsch A.L., Mink J.H. (1993). Prior lateral patellar dislocation: MR imaging findings. Radiology.

[12] Mink J.H., Deutsch A.L. (1989). Occult cartilage and bone injuries of the knee: Detection, classification, and assessment with MR imaging. Radiology.

[13] Shi H., Ding L., Ren S., Jiang Y., Zhang H., Hu X., Huang H., Ao Y. (2021). Prediction of Knee Kinematics at the Time of Noncontact Anterior Cruciate Ligament Injuries Based on the Bone Bruises. Ann. Biomed. Eng..

[14] Moran J., Lee M.S., Kunze K.N., Green J.S., Katz L.D., Wang A., McLaughlin W.M., Gillinov S.M., Jimenez A.E., Hewett T.E. (2023). Examining the Distribution of Bone Bruise Patterns in Contact and Noncontact Acute Anterior Cruciate Ligament Injuries. Am. J. Sports Med..

[15] Tompkins M.A., Rohr S.R., Agel J., Arendt E.A. (2018). Anatomic patellar instability risk factors in primary lateral patellar dislocations do not predict injury patterns: An MRI-based study. Knee Surg. Sports Traumatol. Arthrosc..

[16] Shi H., Ding L., Ren S., Jiang Y., Zhang H., Hu X., Huang H., Ao Y. (2021). Response to the Letter to the Editor on “Prediction of Knee Kinematics at Time of Noncontact Anterior Cruciate Ligament Injuries Based on Bone Bruises”. Ann. Biomed. Eng..

[17] Sanchez-Trigo H., Rittweger J., Sañudo B. (2022). Effects of non-supervised exercise interventions on bone mineral density in adult women: A systematic review and meta-analysis. Osteoporos. Int..

[18] Lin X., Guo H., Lian Y., Kou J., Wang G., Chen Y., Wang J., Han X., Jiang M., Yang Q. (2022). Osteoporosis and Related Health Status Among the Elderly Urban Residents in Elderly-Care Inns in Beijing, a Multicenter DXA Survey. Front. Endocrinol..

[19] Shi H., Ding L., Jiang Y., Zhang H., Ren S., Hu X., Liu Z., Huang H., Ao Y. (2020). Bone Bruise Distribution Patterns After Acute Anterior Cruciate Ligament Ruptures: Implications for the Injury Mechanism. Orthop. J. Sports Med..

[20] Kim-Wang S.Y., Spritzer C.E., Owusu-Akyaw K., Coppock J.A., Goode A.P., Englander Z.A., Wittstein J.R., DeFrate L.E. (2023). The Predicted Position of the Knee Near the Time of ACL Rupture Is Similar Between 2 Commonly Observed Patterns of Bone Bruising on MRI. Am. J. Sports Med..

[21] Green D.W., Perea S.H., Kelly A.M., Potter H.G. (2023). Bone Marrow Edema Injury Patterns in the Pediatric Knee: An MRI Study. HSS J. Musculoskelet. J. Hosp. Spec. Surg..

[22] D’Hooghe P., Grassi A., Villa F.D., Alkhelaifi K., Papakostas E., Rekik R., Marin T., Tosarelli F., Zaffagnini S. (2023). The injury mechanism correlation between MRI and video-analysis in professional football players with an acute ACL knee injury reveals consistent bone bruise patterns. Knee Surg. Sports Traumatol. Arthrosc..

[23] Berger N., Andreisek G., Karer A.T., Bouaicha S., Naraghi A., Manoliu A., Seifert B., Ulbrich E.J. (2017). Association between traumatic bone marrow abnormalities of the knee, the trauma mechanism and associated soft-tissue knee injuries. Eur. Radiol..

[24] Paakkala A., Sillanpää P., Huhtala H., Paakkala T., Mäenpää H. (2010). Bone bruise in acute traumatic patellar dislocation: Volumetric magnetic resonance imaging analysis with follow-up mean of 12 months. Skelet. Radiol..

[25] Cao H., An Q., Gou B., Ma S., Goh E.L., Xiong L., Li Y.G., Ao F. (2019). A new classification of injury patterns of the medial patellofemoral ligament after acute lateral patella dislocation detected using magnetic resonance imaging studies. Injury.

[26] Quatman C.E., Kiapour A., Myer G.D., Ford K.R., Demetropoulos C.K., Goel V.K., Hewett T.E. (2011). Cartilage pressure distributions provide a footprint to define female anterior cruciate ligament injury mechanisms. Am. J. Sports Med..

[27] Hewett T.E., Myer G.D., Ford K.R., Heidt R.S., Colosimo A.J., McLean S.G., van den Bogert A.J., Paterno M.V., Succop P. (2005). Biomechanical measures of neuromuscular control and valgus loading of the knee predict anterior cruciate ligament injury risk in female athletes: A prospective study. Am. J. Sports Med..

[28] Owusu-Akyaw K.A., Kim S.Y., Spritzer C.E., Collins A.T., Englander Z.A., Utturkar G.M., Garrett W.E., DeFrate L.E. (2018). Determination of the Position of the Knee at the Time of an Anterior Cruciate Ligament Rupture for Male Versus Female Patients by an Analysis of Bone Bruises. Am. J. Sports Med..

[29] Richmond C.G., Shea K.G., Burlile J.F., Heyer A.M., Ellis H.B., Wilson P.L., Arendt E.A., Tompkins M.A. (2020). Patellar-Trochlear Morphology in Pediatric Patients from 2 to 11 Years of Age: A Descriptive Analysis Based on Computed Tomography Scanning. J. Pediatr. Orthop..

[30] Diederichs G., Köhlitz T., Kornaropoulos E., Heller M.O., Vollnberg B., Scheffler S. (2013). Magnetic resonance imaging analysis of rotational alignment in patients with patellar dislocations. Am. J. Sports Med..

[31] Patel S.A., Hageman J., Quatman C.E., Wordeman S.C., Hewett T.E. (2014). Prevalence and location of bone bruises associated with anterior cruciate ligament injury and implications for mechanism of injury: A systematic review. Sports Med..

[32] Deangelis J.P., Spindler K.P. (2010). Traumatic Bone Bruises in the Athlete’s Knee. Sports Health.

[33] Agostinone P., Di Paolo S., Lucidi G.A., Fabbro G.D., Grassi A., Zaffagnini S. (2022). Severe bicompartmental bone bruise is associated with rotatory instability in anterior cruciate ligament injury. Knee Surg. Sports Traumatol. Arthrosc..

[34] Sutton K.M., Bullock J.M. (2013). Anterior cruciate ligament rupture: Differences between males and females. J. Am. Acad. Orthop. Surg..

[35] Boden B.P., Sheehan F.T., Torg J.S., Hewett T.E. (2010). Noncontact anterior cruciate ligament injuries: Mechanisms and risk factors. J. Am. Acad. Orthop. Surg..

[36] Fones L., Jimenez A.E., Cheng C., Chevalier N., Brimacombe M.B., Cohen A., Pace J.L. (2021). Trochlear Dysplasia as Shown by Increased Sulcus Angle Is Associated With Osteochondral Damage in Patients With Patellar Instability. Arthroscopy.

[37] Imhoff F.B., Funke V., Muench L.N., Sauter A., Englmaier M., Woertler K., Imhoff A.B., Feucht M.J. (2020). The complexity of bony malalignment in patellofemoral disorders: Femoral and tibial torsion, trochlear dysplasia, TT-TG distance, and frontal mechanical axis correlate with each other. Knee Surg. Sports Traumatol. Arthrosc..

[38] Parikh S.N., Lykissas M.G., Gkiatas I. (2018). Predicting Risk of Recurrent Patellar Dislocation. Curr. Rev. Musculoskelet. Med..

[39] Lyons J.G., Hudson T.L., Krishnamurthy A.B. (2022). Epidemiology of patellar dislocations in the United States from 2001 to 2020: Results of a national emergency department database. Phys. Sport..

[40] Pruneski J., O’Mara L., Perrone G.S., Kiapour A.M. (2022). Changes in Anatomic Risk Factors for Patellar Instability During Skeletal Growth and Maturation. Am. J. Sports Med..

[41] Kanakamedala A.C., Burnham J.M., Pfeiffer T.R., Herbst E., Kowalczuk M., Popchak A., Irrgang J., Fu F.H., Musahl V. (2018). Lateral femoral notch depth is not associated with increased rotatory instability in ACL-injured knees: A quantitative pivot shift analysis. Knee Surg. Sports Traumatol. Arthrosc..

[42] Dimitriou D., Reimond M., Foesel A., Baumgaertner B., Zou D., Tsai T.Y., Helmy N. (2021). The deep lateral femoral notch sign: A reliable diagnostic tool in identifying a concomitant anterior cruciate and anterolateral ligament injury. Knee Surg. Sports Traumatol. Arthrosc..

[43] Berthold D.P., Muench L.N., Herbst E., Mayr F., Chadayammuri V., Imhoff A.B., Feucht M.J. (2021). High prevalence of a deep lateral femoral notch sign in patients with anterior cruciate ligament (ACL) and concomitant posterior root tears of the lateral meniscus. Knee Surg. Sports Traumatol. Arthrosc..

