# Correction: Dai et al. Comparison of Bone Bruise Pattern Epidemiology between Anterior Cruciate Ligament Rupture and Patellar Dislocation Patients—Implications of Injury Mechanism. *Bioengineering* 2023, *10*, 1366

**DOI:** 10.3390/bioengineering12060598

**Published:** 2025-05-31

**Authors:** Ruilan Dai, Yue Wu, Yanfang Jiang, Hongshi Huang, Wenqiang Yan, Huijuan Shi, Qingyang Meng, Shuang Ren, Yingfang Ao

**Affiliations:** 1 Department of Sports Medicine, Peking University Third Hospital, Institute of Sports Medicine of Peking University, Beijing 100080, China; haharuilan@126.com (R.D.); wuyue6063@163.com (Y.W.); anthea_jiang@126.com (Y.J.); huanghs@bjmu.edu.cn (H.H.); 18752009890@163.com (W.Y.); mengqingyang@bjmu.edu.cn (Q.M.); 2 Beijing Key Laboratory of Sports Injuries, Beijing 100080, China; 3 Engineering Research Center of Sports Trauma Treatment Technology and Devices, Ministry of Education, Beijing 100080, China; 4 College of Exercise and Health Sciences, Tianjin University of Sport, Tianjin 300170, China; 5 Biomechanics Laboratory, College of Human Movement Science, Beijing Sport University, Beijing 100080, China; shihuijuan1103@163.com

In the original publication [1], there were mistakes in the published version of Figure 5. There were errors in the percentage results and types of bone bruise patterns of ACL rupture. The corrected Figure 5 appears below:

The authors state that the scientific conclusions are unaffected. This correction was approved by the Academic Editor. The original publication has also been updated.

## Figures and Tables

**Figure 5:**
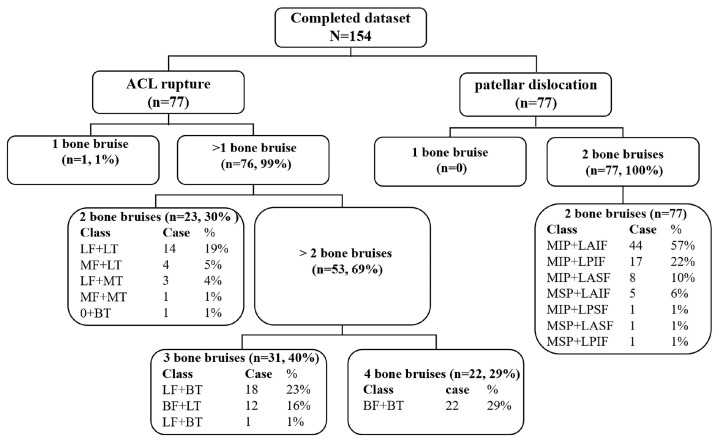
Classification tree of bruise patterns of ACL rupture and patellar dislocation. LF + BT, BF + LT, MF + BT, BF + BT, LF + LT, MF + LT, LF + MT, MF + MT, 0 + BT, 0 + LT, MIP + LAIF, MIP + LPIF, MIP + LASF, MSP + LAIF, MIP + LPSF, MSP + LASF, MSP + LPIF, and the annotation seen above.
